# Ginkgo leaf extract and dipyridamole injection for chronic cor pulmonale: a PRISMA-compliant meta-analysis of randomized controlled trials

**DOI:** 10.1042/BSR20200099

**Published:** 2020-03-12

**Authors:** Jian Qiu, Yijun Guo, Xin Xu, Hongmei Yue, Yapei Yang

**Affiliations:** 1 Department of Cardiology, Liaocheng Third People’s Hospital, Liaocheng 252000, Shandong Province, China; 2 Department of Central Laboratory, Liaocheng People’s Hospital, Liaocheng 252000, Shandong Province, China

**Keywords:** chronic cor pulmonale, conventional treatments, Ginkgo leaf extract and dipyridamole injection, meta-analysis, traditional Chinese medicine

## Abstract

Ginkgo leaf extract and dipyridamole injection (GLED), a kind of Chinese herbal medicine preparation, has been considered as a promising supplementary treatment for chronic cor pulmonale (CCP). Although an analysis of the published literature has been performed, the exact effects and safety of GLED have yet to be systematically investigated. Therefore, a wide-ranging systematic search of electronic databases from which to draw conclusions was conducted. All randomized controlled trials concerning the GLED plus conventional treatments for CCP were selected in the present study. Main outcomes were treatment efficacy, blood gas and hemorrheology indexes, and adverse events. Data from 28 trials with 2457 CCP patients were analyzed. The results indicated that, compared with conventional treatments alone, the combination of conventional treatments with GLED obviously improved the markedly effective rate (RR = 1.44, 95% CI = 1.31–1.58, *P* < 0.00001) and total effective rate (RR = 1.28, 95% CI = 1.18–1.38, *P* < 0.00001). Moreover, the hemorrheology (PaO_2_, *P* < 0.00001; PaCO_2_, *P* < 0.00001; SaO_2_, *P* < 0.00001; pH value, *P* = 0.05) and blood gas indexes (PV, WBHSV, WBMSV, WBLSV, hematocrit and FBG, *P* < 0.01) of CCP patients were also significantly ameliorated after the combined therapy. The frequency of adverse events did not differ significantly between the two groups (*P* > 0.05). In summary, evidence from the meta-analysis suggested that the combination of conventional treatments and GLED appeared to be effective and relatively safe for CCP. Therefore, GLED mediated therapy could be recommended as an adjuvant treatment for CCP.

## Introduction

Chronic cor pulmonale (CCP), a common type of heart disease, has become a rising major public problem that threatens people’s health and quality of life around the world [1]. Although the term “cor pulmonale” is popular in the medical literature, there is presently no consensual definition [1–3]. World Health Organization (WHO) defined CCP as “hypertrophy of the right ventricle resulting from diseases affecting the function and/or structure of the lungs, and may further leading to heart failure” [4]. Pulmonary hypertension resulting from disorders of the respiratory system and/or from chronic hypoxaemia is the main pathological mechanism of CCP [1,5,6]. Currently, the conventional treatment, including antibiotics, vasodilators, expectorants, antiasthmatic drugs, diuretics and antiarrhythic drugs etc. is the main clinical therapy for CCP [1]. However, it is universally acknowledged that long-time use of western medicine sometimes may cause drug resistance and toxic side effects, and therefore its clinical efficacy is still unsatisfactory [1,2]. Many researchers in China and some other Asian countries indicated that the combination of Chinese and Western medicine for CCP might be the potential trend of clinical treatment development in future [7–12].

Ginkgo leaf extract and dipyridamole injection (GLED) is a compound Chinese herbal medicine, which mainly consists of ginkgo flavone glycosides (24–25%), terpene lactones [ginkgolides (3.1%) and bilobalide (2.9%)] and dipyridamole (10%) [13–15]. Ginkgo leaf extract has been proved to be an antioxidant and free radical scavenger, an inhibitor of the platelet-activating factor, a vasodilator, and a regulator of metabolism [14–17]. The therapeutic effect mechanism of GLED on CCP included its ability to scavenge free radicals, reduce inflammation and platelet aggregation while regulating vasodilation and glucose and lipid metabolism [16]. Furthermore, GBE affects vasomotor functions by modulating the synthesis of vasoactive substances including nitric oxide and endothelin [16]. GLED is a combination of Ginkgo leaf extract and dipyridamole (a kind of anti-thrombus and vasodilator drug) [18], and has the pharmacological characteristics of both. GLED has been considered as a promising supplementary treatment option for cardiovascular disease, peripheral vascular disease and pulmonary disease due to its unique biological characteristics [13–17]. Tan et al. [14] reported that GLED (10–40 ml/day per day via intravenous infusion) could relieve the incidence of angina pectoris and improve the hemorheology index of patients with coronary artery disease. Xue et al. [15] showed that the clinical application of GDI (10–40 ml/day per day via intravenous infusion) not only obviously enhanced the overall response rate of conventional treatments, but also effectively improved the blood viscosity and blood lipid level of ischemic stroke patients.

Currently, its application in CCP is garnering much attention [14,19–23]. Several clinical trials reported that conventional treatments combined with GLED exhibits more prominent therapeutic effects for CCP than conventional treatments alone [24–51]. However, the scientific evidence has not been systematically reviewed. In the present study, we conducted a meta-analysis to investigate the clinical efficacy and safety of GLED for CCP, in order to provide the best available evidence for clinical practice and further research planning on CCP treatment.

## Materials and methods

This systematic review and meta-analysis was performed following the PRISMA guidelines and Cochrane Handbook. Ethics approval was not necessary due to the nature of the study (i.e. meta-analysis).

### Search strategy

Literatures were searched across nine electronic databases, including PubMed, Embase, Web of Science, Cochrane Library, Medline, Chinese Scientific Journal Database (VIP), Wanfang database, China National Knowledge Infrastructure (CNKI) and Chinese Biological Medicine Database (CBM), before December 2019, with key terms “ginkgo biloba” or “ginkgo leaf extract” or “ginkgo dipyidamolum” and “dipyridamole injection” or “Ginkgo leaf extract and dipyridamole injection” or “yinxingdamo injection” and “pulmonary heart disease” or “chronic cor pulmonale” or “cor pulmonale” or “fei yuan xing xin zang bing” or “fei xin bing” (Supplementary Table S1). Language is limited with English and Chinese.

### Eligibility criteria

Inclusion criteria:

1. Randomized controlled trials (RCTs) concerning patients diagnosed with CCP were included;
2. Articles involving more than 50 CCP patients;
3. There were no other medicines in combination with the conventional treatments in the experimental group, except for GLED, compared with the conventional treatments as a control;
4. One or more outcome measures, including the therapeutic effect, or hemorheology or blood gas indexes, or adverse events must be included in each study.

Exclusion criteria:

1. Studies not focus on GLED were excluded;
2. Inappropriate criteria in experimental or control group were excluded;
3. Articles without sufficient available data were excluded;
4. Non-RCTs, literature reviews, meta-analysis, meeting abstracts and case reports, repeated studies and experimental model researches were excluded.

### Data extraction and quality assessment

Data were extracted by two reviewers (Guo, Y. J. and Xu, X.) independently according to the same inclusion and exclusion criteria; disagreements were adjudicated by the third investigator (Yue, H. M.). The extracted characteristics comprised the following items: (a) first authors’ names, (b) years of publication, (c) NYHA heart function classification, (d) number of cases, (e) patient ages, (f) intervening measure, (g) dosage of GLED, (h) duration of treatment, (i) manufacturer of GLED and (j) study parameter types. The quality of included trials was evaluated according to Cochrane Handbook [52,53].

### Outcome definition

Treatment efficacy was evaluated in terms of markedly effective rate (MER) and the total effective rate (TER), blood gas and hemorrheology indexes. The hemorrheology indexes covered the following indicators: plasma viscosity (PV), whole blood high-shear viscosity (WBHSV), whole blood medium-shear viscosity (WBMSV), whole blood low-shear viscosity (WBLSV), hematocrit, erythrocyte aggregation index (EAI) and content of fibrinogen (FBG). The blood gas indicators [partial pressure of oxygen (PaO_2_), partial pressure of carbon dioxide (PaCO_2_), saturation of hemoglobin with oxygen (SaO_2_) and pH value] of CCP patients were also determined and compared between the GLED and non- GLED groups.

### Statistical analysis

Statistical analysis was performed using the Review Manager 5.3 (Nordic Cochran Centre, Copenhagen, Denmark) and Stata 13.0 (Stata Corp., College Station, TX, U.S.A.) statistical software. Dichotomous data were represented by the risk ratio (RR) with the respective 95% confidence interval (CI), whereas continuous variables were expressed as mean difference (MD) with 95% CI. *P* < 0.05 indicates difference with statistical significance. Cochrane’s *Q*-test and *I*^2^ statistics were used to assess heterogeneity between studies; *P* < 0.1 or *I*^2^ > 50% indicates statistical heterogeneity [54]. A fixed-effects model was used to pool the estimates when heterogeneity was absent. Otherwise, a random effects model was selected.

Publication bias was evaluated by Begg’s and Egger’s regression tests [55]. If publication bias existed, a trim-and-fill method should be applied to coordinate the estimates from unpublished studies, which were compared with the original pooled RR [56]. Sensitivity analysis was conducted to investigate the influence of different GLED dosages, duration of treatment, sample sizes of involved studies, and manufacturer of GLED on clinical efficacy.

## Results

### Search results

**Figure 1:**
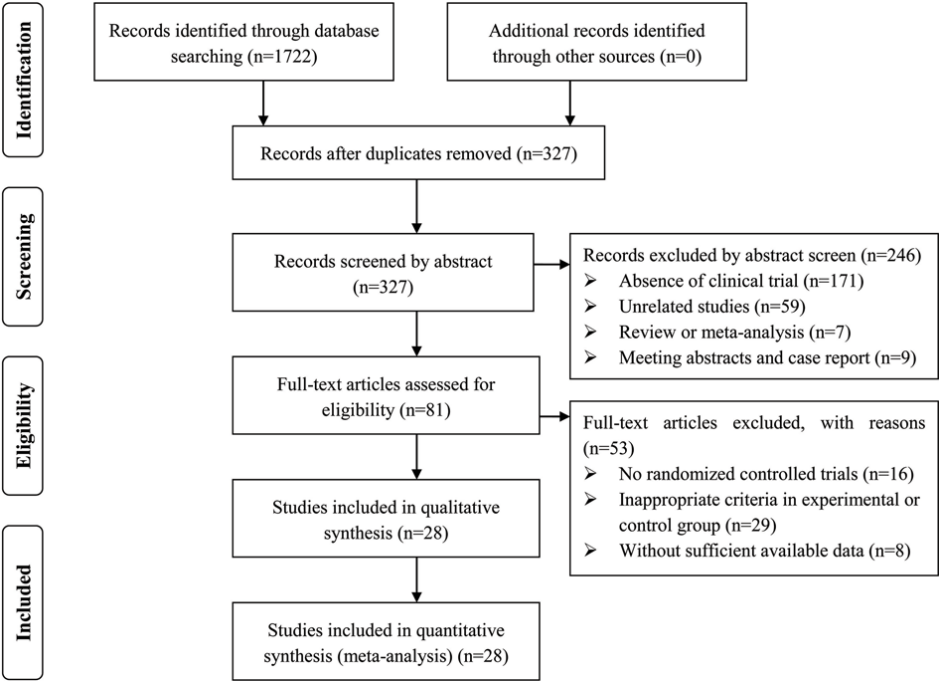
Study selection process for the meta-analysis

A total of 1722 articles were identified with initial retrieve. A total of 1395 papers were excluded due to duplication. After title and abstract review, 246 articles were further excluded because they were not clinical trials (*n* = 171) or were unrelated studies (*n* = 59) or were reviews and meta-analysis (*n* = 7) or were meeting abstracts and case report (*n* = 9), leaving 81 studies as potentially relevant. After detailed assessment of full texts, articles were not RCTs (*n* = 16), publications with inappropriate criteria of experimental or control group (*n* = 29) and trials with insufficient data (*n* = 8) were excluded. Finally, 28 trials [24–51] involving 2457 CCP patients were included in this analysis (Figure 1).

### Patient characteristics

After selection, all included trials were performed in different hospital of China. In total, 1214 CCP patients were treated by conventional treatments in combination with GLED adjuvant therapy, while 1243 patients were treated by conventional treatments alone. Detailed information of the involved studies and CCP patients is shown in Table 1. All included trials except two [36,43] clearly introduce the duration of treatment. Fourteen studies [24–31,34,35,41,45,46,50] specifically describe the manufacturer of GLED and the remaining 14 studies [32,33,36–40,42–44,47–49,51] lacked clear description of production information (Supplementary Table S2).

**Table 1:** Clinical information from the eligible trials in the meta-analysis

Included studies	NYHA classification	Patients Con/Exp	Age (year)		Intervening measure (Exp/Con)	Dosage of GLED	Duration
			Con	Exp			
Fan, J., 2011	II-IV	40/40	63 ± 5.6 (mean)	61 ± 7.8 (mean)	CT+GLED (iv) VS CT	20 ml/day	14 days
Gan, L., 2015	II-IV	43/43	67.00 ± 1.48 (mean)	68.50 ± 1.20 (mean)	CT+GLED (iv) VS CT	20 ml/day	10–-14 days
Gao, L. S., 2006	NG	48/48	NG	NG	CT+GLED (iv) VS CT	20 ml/day	7 days
Gao, L. Y., 2009	NG	40/40	58 ± 11 (mean)	59 ± 11 (mean)	CT+GLED (iv) VS CT	20 ml/day	14 days
He, F. Z., 2009	III-IV	35/36	46–78 (range)	45–80 (range)	CT+GLED (iv) VS CT	20 ml/day	14 days
He, H., 2019	NG	31/31	68 ± 9 (mean)	68 ± 10 (mean)	CT+GLED (iv) VS CT	20 ml/day	12 days
He, K. X., 2012	III-IV	31/31	62.41 ± 3.52 (mean)	62.36 ± 3.61 (mean)	CT+GLED (iv) VS CT	20 ml/day	7–14 days
Hu, Z. W., 2013	NG	40/40	59.2 ± 5.6 (mean)	60.2 ± 5.2 (mean)	CT+GLED (iv) VS CT	20 ml/day	10 days
Jia, X. H., 2009	II-IV	33/33	41–82 (range)	42–78 (range)	CT+GLED (iv) VS CT	40 ml/day	15 days
Ji, N. P., 2010	II-IV	48/58	58–89 (range)	60–91 (range)	CT+GLED (iv) VS CT	20 ml/day	10 days
Liang, Y. M., 2007	III-IV	48/48	NG	NG	CT+GLED (iv) VS CT	30 ml/day	14 days
Liu, L. Q., 2012	NG	64/64	64.5 ± 8.9 (mean)	63.8 ± 8.6 (mean)	CT+GLED (iv) VS CT	20 ml/day	10 days
Liu, R. P., 2009	II-IV	31/30	50–79 (range)	51–79 (range)	CT+GLED (iv) VS CT	15-20 ml/day	NG
Li, W. M., 2009	NG	51/52	49–92 (range)	45–95 (range)	CT+GLED (iv) VS CT	20 ml/day	14 days
Li, X. D., 2016	III-IV	45/45	60.89 ± 3.87 (mean)	61.02 ± 3.76 (mean)	CT+GLED (iv) VS CT	20 ml/day	7–14 days
Tao, L., 2015	II-IV	54/54	NG	NG	CT+GLED (iv) VS CT	30 ml/day	14 days
Wang, B. C., 2011	III-IV	28/28	63.1 ± 3.9 (mean)	62.2 ± 4.3 (mean)	CT+GLED (iv) VS CT	20 ml/day	15 days
Wang, L. H., 2014	II-IV	56/56	71.8 ± 6.5 (mean)	72.5 ± 6.9 (mean)	CT+GLED (iv) VS CT	20 ml/day	10 days
Wang, Y., 2017	II-III	36/36	NG	NG	CT+GLED (iv) VS CT	20 ml/day	10 days
Xie, J., 2012	NG	50/50	67.0 ± 6.5 (mean)	68.0 ± 7.0 (mean)	CT+GLED (iv) VS CT	20 ml/day	NG
Xu, C. H., 2008	NG	40/46	55.4 ± 7.8 (mean)	58.6 ± 7.3 (mean)	CT+GLED (iv) VS CT	15 ml/day	7 days
Yang, J. L., 2010	III-IV	30/30	NG	NG	CT+GLED (iv) VS CT	20 ml/day	7–10 days
Yang, Y. P., 2011	III-IV	35/35	41.5–79 (range)	40–80 (range)	CT+GLED (iv) VS CT	20 ml/day	14 days
Yin, Y. W., 2008	II-IV	29/36	38–83 (range)	40–81 (range)	CT+GLED (iv) VS CT	40 ml/day	15 days
Zhong, G. N., 2015	NG	60/60	68.36 ± 5.24 (mean)	68.41 ± 5.33 (mean)	CT+GLED (iv) VS CT	20 ml/day	12 days
Zhou, B., 2012	III-IV	43/43	56–72 (range)	58–76 (range)	CT+GLED (iv) VS CT	25 ml/day	28 days
Zhou, C. Y., 2015	NG	25/30	NG	NG	CT+GLED (iv) VS CT	20-30 ml/day	10 days
Zou, D.H., 2009	II-IV	100/100	40–92	38–94	CT+GLED (iv) VS CT	20 ml/day	days

**Notes:** Con, control group (conventional treatments alone group); Exp, experimental group (conventional treatments and GLED combined group).

*The compositions and concentrations of GLED in all included trials are the same (every 10 ml GDI contained 9.0–11.0 mg total flavonoids and 3.6–4.4 mg dipyridamole).

**Abbreviations:** CT, conventional treatments; NG, not given; NYHA, New York Heart Association; GLED, Ginkgo leaf extract and dipyridamole injection; IV, intravenous injection.

### Quality assessment

**Figure 2:**
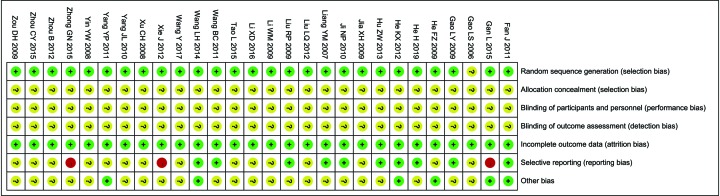
Risk of bias summary Review of authors’ judgments about each risk of bias item for included studies. **Note:** Each color represents a different level of bias: red for high-risk, green for low-risk, and yellow for unclear-risk of bias.

The assessment of bias risk is shown in Figure 2. Twenty-seven studies were determined as low risk, while one trial [26] did not provide a clear description of the randomization process. All included trials did not provide clear description of performance and detection risks. The attrition risks of involved trials were low. Three trials [25,43,48] were considered as high reporting risk owing to lack of primary outcomes (MER or TER) and 15 studies [25,26,28,32,35,37–39,42–51] were considered as unclear reporting risk due to lack of safety assessment.

### TER and MER

**Figure 3:**
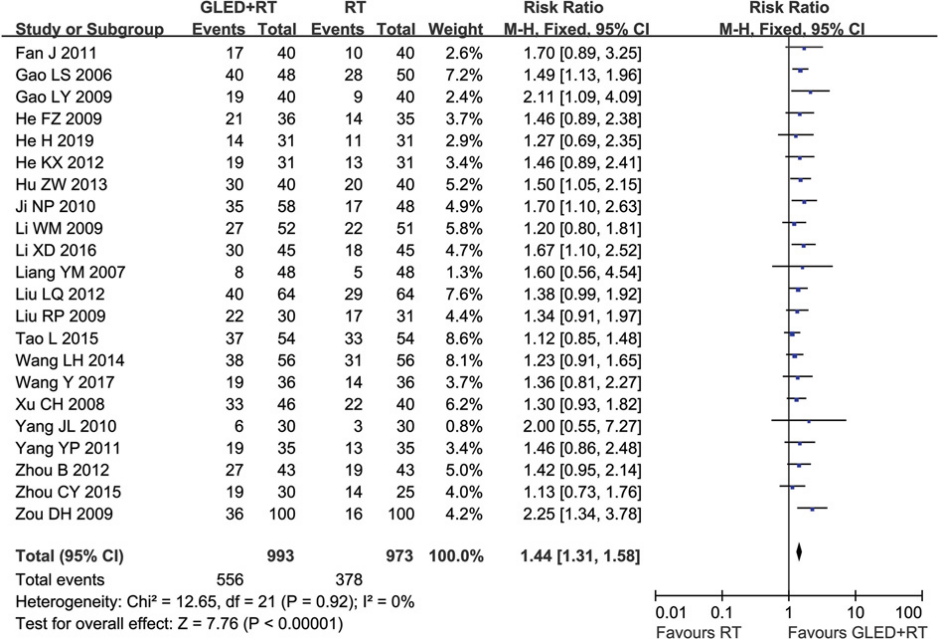
Comparisons of MER between experimental and control group Forest plot of the comparison of MER between the experimental and control group. Control group, conventional treatments alone group; Experimental group, conventional treatments and GLED combined group. The random effects meta-analysis model (Inverse Variance method) was used.

**Figure 4:**
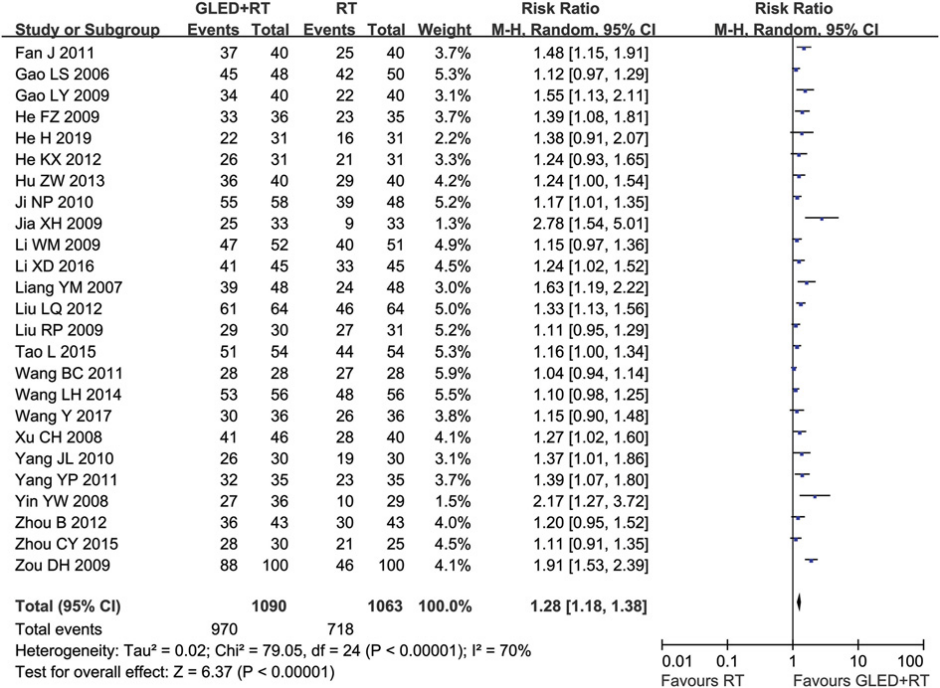
Comparisons of TER between experimental and control group Forest plot of the comparison of TER between the experimental and control group. Control group, conventional treatments alone group; Experimental group, conventional treatments and GLED combined group. The random effects meta-analysis model (Mantel–Haenszel method) was used.

Twenty-five clinical trials [24,26–42,44–47,49–51] involving 2,153 cases compared the MER and/or TER between the two groups (Figures 3 and 4). Our pooled results showed that CCP patients underwent combined therapy had significantly increased MER (RR = 1.44, 95% CI = 1.31–1.58, *P* < 0.00001) and TER (RR = 1.28, 95% CI = 1.18–1.38, *P* < 0.00001) compared with conventional treatments alone. MER (*P* = 0.92, *I*^2^ = 0%) was not heterogeneous among the studies, so fixed-effect model was used to analyzing its RR. Otherwise, random-effect model was used.

### Blood gas analysis

**Figure 5:**
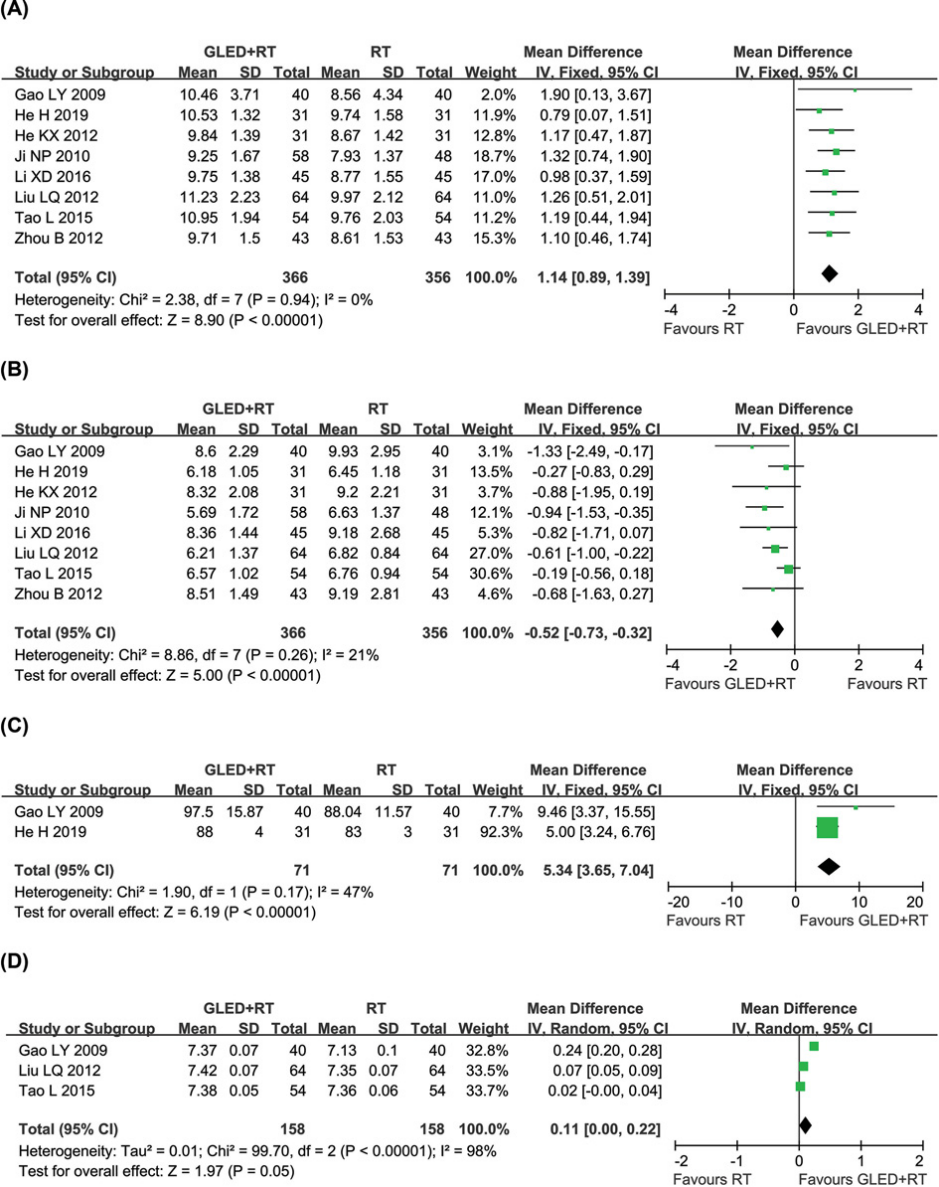
Comparisons of blood gas indexes between experimental and control group Forest plot of the comparison of the blood gas indexes including PaO_2_ (**A**), PaCO_2_ (**B**), SaO_2_ (**C**) and PH value (**D**) between the experimental and control group. Control group, conventional treatments alone group; Experimental group, conventional treatments and GLED combined group.

Eight trials [27,29,30,33,35,38,39,49] with 722 participants measured the PaO_2_ and PaCO_2_, two trials [27,29] involving 142 CCP patients evaluated the SaO_2_, and three trials [27,35,39] including 316 patients reported data on pH value (Figure 5). Results showed that the blood gas indexes of CCP patients received combined therapy was obviously improved compared with those treated by conventional treatments alone, indicated by significantly increased PaO_2_ (MD = 1.14, 95% CI = 0.89–1.39, *P* < 0.00001)_,_ SaO_2_ (MD = 5.34, 95% CI = 3.65–7.04, *P* < 0.00001) and PH value (MD = 0.11, 95% CI = 0.00–0.22, *P* = 0.05), and obviously decreased PaCO_2_ (MD = -0.52, 95% CI = -0.73–0.32, *P* < 0.00001). PH value (*P* = 0.99, *I*^2^ = 0%) was heterogeneous among the studies, so random-effect model was used to analyzing its RR. Otherwise, fixed-effect model was used.

### Hemorrheology assessment

**Figure 6:**
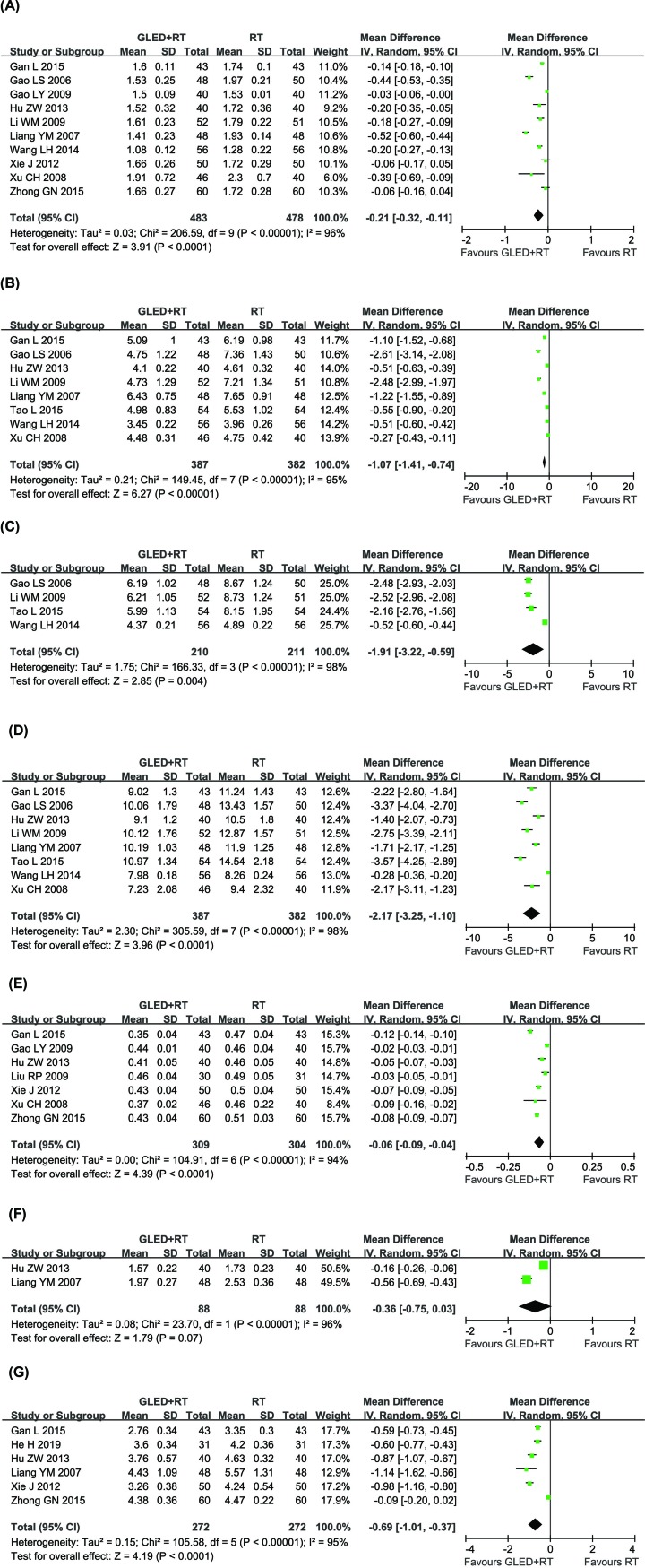
Comparisons of hemorrheology indexes between experimental and control group Forest plot of the comparison of the hemorrheology indexes including PV (**A**), WBHSV (**B**), WBMSV (**C**), WBLSV (**D**), hematocrit (**E**), EAI (**F**) and FBG (**G**) between the experimental and control group. Control group, conventional treatments alone group; Experimental group, conventional treatments and GLED combined group. The random effects meta-analysis model (Mantel–Haenszel method) was used.

The hemorrheology of CCP patients was measured between GLED and non-GLED groups in 13 controlled studies [25–27,29,31,34,36,37,39,41,43,44,48] with 1,192 CCP patients (Figure 6). In this analysis, our results showed that the hemorrheology of CCP patients received combined therapy was significantly ameliorated compared with those treated by conventional treatments alone, indicated by significantly decreased PV (MD = -0.21, 95% CI = -0.32–0.11, *P* < 0.0001), WBHSV (MD = -1.07, 95% CI = -1.41–0.74, *P* < 0.00001), WBMSV (MD = -1.91, 95% CI = -3.22–0.59, *P* = 0.004), WBLSV (MD = -2.17, 95% CI = -3.25–1.10, *P* < 0.0001), hematocrit (MD = -0.06, 95% CI = -0.09–0.04, *P* < 0.0001) and FBG (MD = -0.69, 95% CI = -1.01–0.37, *P* < 0.0001), whereas analysis of EAI (MD = -0.36, 95% CI = -0.75-0.03, *P* = 0.07) did not differ significantly between the two groups. There was significant heterogeneity among the studies. Therefore, a random-effects model was conducted to pool data and so any conclusions need to be made with caution.

### Adverse events assessment

**Figure 7:**
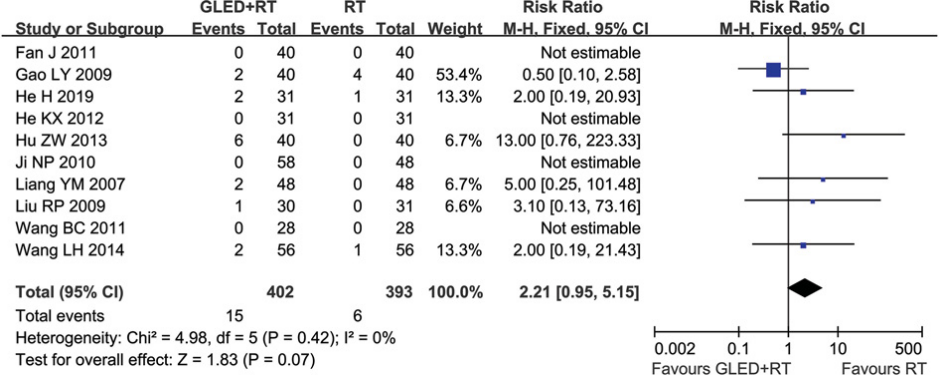
Comparisons of total adverse effects between experimental and control group Forest plot of the comparison of total adverse effects between the experimental and control group. Control group, conventional treatments alone group; Experimental group, conventional treatments and GLED combined group. The fixed-effects meta-analysis model (Mantel–Haenszel method) was used.

Among all included studies, 18 trials [25,26,28,32,35,37–39,42–51] did not report adverse events. Ten trials [24,27,29–31,33,34,36,40,41] involving 795 CCP patients described specific adverse events that occurred in GLED treatment. The most common side effects of GLED treatment were including nausea, headache, dizziness, abdominal distention, pruritus and skin rash, which usually subsided after symptomatic treatment. No severe adverse event occurred during GLED treatment, and the occurrence of these adverse reactions in the two groups did not differ obviously (Figure 7, RR = 2.21, 95% CI = 0.95–5.15, *P* = 0.07). Statistics showed no statistically significant heterogeneity (*P* = 0.42, *I*^2^ = 0%), so fixed-effect model was used to carry out the meta-analysis.

### Publication bias

**Figure 8:**
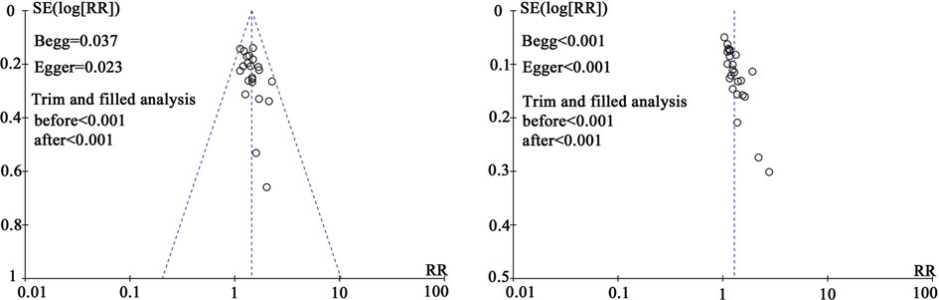
Funnel plot of MER (A) and TER (B)

Publication bias was assessed by funnel plots, Begg’s and Egger’s regression tests (Figure 8). Analysis results indicate that publication bias was existed in MER and TER. To determine if the bias affect the results of pooled analysis, we conducted trim and filled analysis. The adjusted RR indicated same trend with the result of the primary analysis (Figure 8, MER: before: *P* < 0.001, after: *P* < 0.001; TER: before: *P* < 0.001, after: *P* < 0.001), reflecting the reliability of our primary conclusions.

### Sensitivity analysis

**Figure 9:**
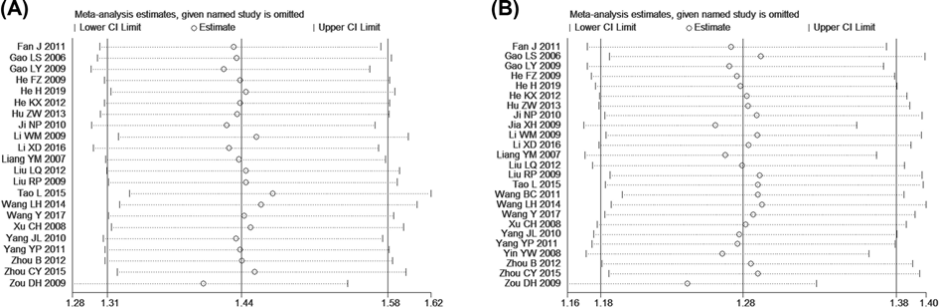
Sensitivity analysis for MER (A) and TER (B)

Sensitivity analysis was performed to explore an individual study’s influence on the pooled results by deleting one single study each time from pooled analysis. As Figure 9 signified, the results revealed that no individual studies significantly affected the primary indicators (MER and TER), which indicated statistically robust results.

We also conducted subgroup analysis to explore the source of heterogeneity in MER and TER with respect to GLED dosages, duration of treatment, sample sizes of involved studies, and manufacturer of GLED. As shown in Table 2, our analysis showed that these variables except manufacturer of GLED did not have a significant impact on the therapeutic efficacy of GLED for CCP.

**Table 2:** Subgroup analyses of MER and TER between the experimental and control group

Parameter	Factors at study level	Experimental group, No. of patients (*n*)	Control group, No. of patients (*n*)	Analysis method	Heterogeneity		Risk ratio (RR)	95% CI	*P*-value
					*I*^2^ (%)	*P*-value			
MER	**Dosage of GLED (DG)**								
	20< DG ≤ 40 ml/day	189	188	Fixed	16	0.31	1.32	1.08–1.61	0.006
	15≤ DG ≤ 20 ml/day	774	760	Fixed	0	0.98	1.49	1.34–1.66	<0.00001
	**Duration of treatment**								
	≥14 days	364	362	Fixed	0	0.70	1.38	1.19–1.60	<0.0001
	<14 days	520	501	Fixed	0	0.69	1.48	1.31–1.68	<0.00001
	**Manufacturer of the GLED**								
	I	70	70	Fixed	0	0.82	1.77	0.99–3.16	0.05
	II	92	91	Fixed	0	0.42	1.34	1.02–1.76	0.03
	III	322	323	Fixed	0	0.87	1.47	1.26–1.70	<0.00001
	IV	30	25	Fixed			1.13	0.73–1.76	0.58
	**Study sample size**								
	>80	596	582	Fixed	0	0.53	1.42	1.27–1.58	<0.00001
	≤80	397	391	Fixed	0	0.97	1.48	1.25–1.75	<0.00001
TER	**Dosage of GLED**								
	20< DG ≤ 40 ml/day	283	265	Fixed	49	0.08	1.27	1.16–1.39	<0.00001
	15≤ DG ≤ 20 ml/day	777	773	Random	76	<0.00001	1.30	1.18–1.44	<0.00001
	**Duration of treatment**								
	≥14 days	486	467	Random	69	0.0004	1.27	1.14–1.41	<0.0001
	<14 days	495	486	Random	74	<0.0001	1.29	1.14–1.48	0.0001
	**Manufacturer of the GLED**								
	I	70	70	Fixed	0	0.70	1.43	1.18–1.74	0.0003
	II	92	91	Fixed	0	0.59	1.19	1.04–1.36	0.01
	III	322	323	Fixed	31	0.18	1.26	1.16–1.36	<0.00001
	IV	30	25	Fixed			1.11	0.91–1.35	0.29
	**Study sample size**								
	>80	519	496	Random	67	0.0001	1.30	1.16–1.45	<0.00001
	≤80	571	567	Random	75	<0.0001	1.26	1.13–1.40	<0.0001

**Notes:** Con, control group (conventional treatments alone group); Exp, experimental group (conventional treatments and GLED combined group); I, Shanxi Pude Pharmaceutical Co., Ltd.; II, Hubei Minkang Pharmaceutical Co., Ltd.; III, Guizhou Yibai Pharmaceutical Co., Ltd.; IV, Tonghua Guhong Pharmaceutical Co., Ltd.

**Abbreviations:** GLED: Ginkgo leaf extract and dipyridamole injection; MER: Markedly effective rate; TER: Total effective rate.

## Discussion

Ginkgo biloba, a “living fossil,” have been used as traditional herbal medicine for thousands of years in China [16]. As an important Ginkgo biloba extract preparation, it has been proven that the pharmacological effects of GLED include regulating vasomotor, improving hemorheology, enhancing immunity, relieving inflammation and scavenging free radicals [14,15,57]. It has been clinically applied as an effective complementary drug for lung and heart disease [14,15,58–60]. Even though there was statistical analysis of published literatures, a comprehensive and systematic evaluation of GLED for the treatment of CCP is still rare. In this analysis, we conducted a wide range of online search according strict eligibility criteria, by which to provide an internationally accessible systematic review of the clinical efficacy and safety of GLED for the CCP.

The meta-analysis was carried out in 25 articles [24,26–42,44–47,49–51] to evaluate the therapeutic effects of GLED for CCP. Compared with conventional treatments alone, GLED combined with conventional treatments was associated with obviously higher MER and TER. Moreover, because of chronic hypoxia, patients with CCP will suffer from oxygen free radicals metabolism imbalance, increased blood viscosity and pulmonary artery pressure, and further lead to the right heart dysfunction and even failure [1–4]. Our analysis results showed that the blood gas and hemorheology indexes of patients were also significantly ameliorated after conventional treatments and GLED combined therapy. All these results indicated that GLED can improve the cardiopulmonary function of CCP patients effectively, which may be related with its action on regulating the blood viscosity and blood gas indexes. To further confirm whether some variable factors affect the therapeutic effects of GLED for CCP. We used four clinical variables (GLED dosages, duration of treatment, sample sizes of involved studies, and manufacturer of GLED) to interact with two outcome indicators (MER and TER) and found that the MER and TER might be associated with manufacturer of GLED. However, given that only one study [50] reported the use of GLED produced by Tonghua Guhong Pharmaceutical Co., Ltd., therefore it is not enough to draw a definitive conclusion at present.

Safety is the top priority of a therapeutic strategy, and special attention should be paid to adverse drug events. The most common side effects during GLED therapy were nausea, headache, dizziness, abdominal distention, pruritus and skin rash, and the total adverse events did not differ significantly between the two groups. Therefore, GLED is a relatively safe auxiliary medicine for CCP. However, evidence was limited to make a conclusion on safety evaluation because only 10 studies mentioned the adverse events.

There are some limitations in our analysis. First, as an important Chinese herb preparation, GLED was mainly applied in China or other Asian countries, which may bring the unavoidable regional bias and subsequently influence the clinical application of GLED worldwide. Second, the duration of treatment ranged from 7 to 28 days among the included studies. All of the trials assessed the efficacy immediately after the termination of the treatment period. Therefore, the long-term effect of GLED for CCP still needs methodologically rigorous trials to verify. Third, the main pathological features of CCP are pulmonary artery pressure, progressive of right ventricular hypertrophy and cardiopulmonary functional insufficiency. Many key variables, such as number of exacerbation, heart and lung function, quality of life, which closely related to survival, are not evaluated in these studies. Therefore, it is not yet possible to draw a conclusion on important outcomes. Moreover, GLED is a mixture that consists of more than one effective component, in this case, the mechanism of GLED may be complex and has multiple targets, and also this limits the usage of it. Fourthly, the administration conditions (extract/distill methods, storage conditions, dripping speed, GLED dosages and manufacturer, et al.) might be related with the efficacy of GLED mediated therapy [61,62]. In order to achieve the clinical therapeutic effect and reduce the incidence of adverse reactions of GLED mediated therapy to the greatest extent, it is necessary to strengthen the supervision of clinical medication to standardize the rational medication. The GLED in all included trials was approved by Chinese State Food and Drug Administration (SFDA), and granted the Manufacturing Approve Number issued by Chinese SFDA (Supplementary Table S2). Based on currently available literatures, we have conducted subgroup analysis according to different GLED dosages and manufacturer. However, there are insufficient data to perform a statistical analysis to evaluate the impacts of other variable factors (extract/distill methods, storage conditions and dripping speed, et al.) on the treatment effect of GLED. We will keep following up with upcoming clinical trials to obtain relevant data when available. Finally, allocation concealment and blind method were not clear in most included studies, which may results in exaggerated estimates of treatment effect. Given the limitations mentioned above, all the findings from our study should be dealt with some caution.

## Conclusion and prospect

In summary, our meta-analysis suggested that GLED could have potential therapeutic effects and be relatively safe for the treatment of CCP. Clinical application of GLED not only obviously enhanced the therapeutic effects of conventional treatments, but also useful in lowering plasma viscosity, blood viscosity, hematocrit, alleviating and improving PaO_2_ and SaO_2_ of CCP patients. However, due to the publication bias and low quality of some included trials increases risks and bias, the clinical efficacy and safety of GLED-mediate therapy for CCP still needs more high-quality, multi-center large randomized trials to verify.

Traditional Chinese medicine plays an increasingly important role in various disease treatments (such as malaria and 2019 novel coronavirus, etc.) [16,63]. Ginkgo leaf extract has been used for pharmaceutical and medicinal purpose in China for several hundred years to treat various diseases [16]. The extremely low rate of side effects and good tolerance together with its pharmacological mechanism will make GLED a promising therapeutic drug in cardiovascular disease, peripheral vascular disease and pulmonary disease worldwide [16].

## Supplementary Material

Supplementary Tables S1-S3

## Abbreviations

CCP: chronic cor pulmonale
CI: confidence interval
EAI: erythrocyte aggregation index
FBG: content of fibrinogen
GLED: Ginkgo leaf extract and dipyridamole injection
MD: mean difference
MER: markedly effective rate
NYHA: New York Heart Association
PaCO_2_: partial pressure of carbon dioxide
PaO_2_: partial pressure of oxygen
PRISMA: Preferred Reporting Items for Systematic Reviews
PV: plasma viscosity
RCT: randomized controlled trial
RR: risk ratio
SaO_2_: saturation of hemoglobin with oxygen
TER: total effective rate
WBHSV: whole blood high-shear viscosity
WBLSV: whole blood low-shear viscosity
WBMSV: whole blood medium-shear viscosity
WHO: World Health Organization
