# Supplementary material for: Ginkgo leaf extract and dipyridamole injection for chronic cor pulmonale: a PRISMA-compliant meta-analysis of randomized controlled trials
Source: Biosci Rep. 2020 Mar 12;40(3):BSR20200099. doi: 10.1042/BSR20200099 (PMC7069918; doi:10.1042/BSR20200099)
Supplement: Supplementary Tables S1-S3 [file BSR-2020-0099_supp.pdf]

Supplement Table 1. Searching strategy for electronic databases

**Data base**	**Search Strategy**
**English database:** PubMed, Embase, Web of Science, Cochrane Library and Medline	**#1.** “pulmonary heart disease” or “chronic pulmonary heart disease” or “chronic cor pulmonale” or “cor pulmonale” [Title/Abstract].<br>**#2.** “cor pulmonale” [MeSH].<br>**#3.** #1 or #2.<br>**#4.** “ginkgo biloba” or “ginkgo biloba extract” or “ginkgo leaf extract” or “yinxingdamo” or “ginkgo dipyidamolum” or “ginkgo leaf extract and dipyridamole” or “yinxing” or “yinxingtiquwu” [Title/Abstract].<br>**#5.** “injection”.<br>**#6. #3** and **#4** and **#5**<br>**#7.** limit **#6** to human<br>**#8.** limit **#7** to (Randomized controlled clinical trial)<br>**#9.** limit **#8** to yr="2000- December 2019"

**Chinese database:** Chinese Scientific Journal Database (VIP), Wanfang database, China National Knowledge Infrastructure (CNKI) and Chinese Biological Medicine Database (CBM)	**#1.** “fei xin bing” or “fei yuan xing xin zang bing” or “fei xin bing xin shuai” or “ fei xin bing xin li shuai jie” or “fei xin bing xin gong neng bu quan” or “fei xin bing xin gong neng shuai jie” or “fei yuan xing xin zang bing xin shuai” or “fei yuan xing xin zang bing xin li shuai jie” or “fei yuan xing xin zang bing xin gong neng bu quan” or “fei yuan xing xin zang bing xin gong neng shuai jie” [Title/Keywords].<br>**#2.** “yin xing” or “yin xing ti qu wu” or yin xing ye ti qu wu” or “yinxingdamo” [Title/Keywords].<br>**#3.** “zhu she ye” or “zhu she ji” [Title/Keywords].<br>**#4. #1** and **#2** and **#3**.<br>**#5.** limit **#4** to human<br>**#6.** limit **#7** to (Randomized controlled clinical trial)<br>**#7.** limit **#8** to yr="2000-December 2019"

Supplement Table 2. Information of GLED combined with conventional treatments

Included studies	Manufacturer	Manufacturing Approve Number	Enrollment Period	Parameter types
Fan J 2011	Shanxi Pude Pharmaceutical Co., Ltd.	H14023516	2008.11-2010.8	MER, TER, AE
Gan L 2015	Guizhou Yibai Pharmaceutical Co., Ltd.	H52020032	2010.1-2013.7	HA
Gao LS 2006	Guizhou Yibai Pharmaceutical Co., Ltd.	H52020032	Not given	MER, TER, HA
Gao LY 2009	Guizhou Yibai Pharmaceutical Co., Ltd.	H52020032	2006.3-2008.8	MER, TER, BGA, HA, AE
He FZ 2009	Guizhou Yibai Pharmaceutical Co., Ltd.	H52020032	2004.12-2008.8	MER, TER,
He H 2019	Guizhou Yibai Pharmaceutical Co., Ltd.	H52020032	2015.6-2018.6	MER, TER, BGA, HA, AE
He KX 2012	Guizhou Yibai Pharmaceutical Co., Ltd.	H52020032	2008.4-2012.4	MER, TER, BGA, AE
Hu ZW 2013	Hubei Minkang Pharmaceutical Co., Ltd.	H42022869	2010.1-2012.1	MER, TER, HA, AE
Jia XH 2009	Not given	Not given	2006.6-2009.1	TER,
Ji NP 2010	Not given	Not given	2009.6-2010.4	MER, TER, BGA, AE
Liang YM 2007	Hubei Minkang Pharmaceutical Co., Ltd.	H42022869	Not given	MER, TER, HA, AE
Liu LQ 2012	Guizhou Yibai Pharmaceutical Co., Ltd.	H52020032	2011.3-2012.8	MER, TER, BGA
Liu RP 2009	Not given	Not given	Not given	MER, TER, HA , AE
Li WM 2009	Not given	Not given	2005.3-2008.2	MER, TER, HA
Li XD 2016	Not given	Not given	2016.3-2017.3	MER, TER, BGA
Tao L 2015	Not given	Not given	2011.12-2014.12	MER, TER, BGA, HA
Wang BC 2011	Not given	Not given	2009.6-2009.11	TER, AE
Wang LH 2014	Guizhou Yibai Pharmaceutical Co., Ltd.	H52020032	2011-2013	MER, TER, HA, AE

Wang Y 2017	Not given	Not given	2015.1-2016.1	MER, TER,
Xie J 2012	Not given	Not given	2008.9-2011.2	HA
Xu CH 2008	Not given	Not given	2005.1-2007.6	MER, TER, HA
Yang JL 2010	Shanxi Pude Pharmaceutical Co., Ltd.	H14023516	1998-1999	MER, TER,
Yang YP 2011	Guizhou Yibai Pharmaceutical Co., Ltd.	H52020032	2005.10-2010.10	MER, TER,
Yin YW 2008	Not given	Not given	2006.1-2008.1	TER,
Zhong GN 2015	Not given	Not given	2012.7-2013.12	HA
Zhou B 2012	Not given	Not given	2010.1-2011.4	MER, TER, BGA
Zhou CY 2015	Tonghua Guhong Pharmaceutical Co., Ltd.	H22026140	2012-2014	MER, TER,
Zou DH 2009	Not given	Not given	2002.1-2007.12	MER, TER,

Abbreviations: GLED: Ginkgo leaf extract and dipyridamole injection; MER: Markedly effective rate; TER: Total effective rate; BGA: Blood gas analysis; HA: Hemorrheology Assessment; AE: adverse events.

Supplement Table 3. PRISMA 2009 checklist

Section/topic	#	Checklist item	Reported on page #
**TITLE**			
Title	1	Identify the report as a systematic review, meta-analysis, or both.	Page 1 (Title)
**ABSTRACT**			
Structured summary	2	Provide a structured summary including, as applicable: background; objectives; data sources; study eligibility criteria, participants, and interventions; study appraisal and synthesis methods; results; limitations; conclusions and implications of key findings; systematic review registration number.	Page 2 (Abstract)
**INTRODUCTION**			
Rationale	3-4	Describe the rationale for the review in the context of what is already known.	Page 3-4 (Introduction)
Objectives	N/A	Provide an explicit statement of questions being addressed with reference to participants, interventions, comparisons, outcomes, and study design (PICOS).	N/A
**METHODS**			
Protocol and registration	N/A	Indicate if a review protocol exists, if and where it can be accessed (e.g., Web address), and, if available, provide registration information including registration number.	N/A
Eligibility criteria	5-6	Specify study characteristics (e.g., PICOS, length of follow-up) and report characteristics (e.g., years considered, language, publication status) used as criteria for eligibility, giving rationale.	Page 5-6 (Search strategy and selection criteria,

			Supplementary Table 1)
Information sources	5-6	Describe all information sources (e.g., databases with dates of coverage, contact with study authors to identify additional studies) in the search and date last searched.	Page 5-6 (Search strategy and selection criteria, Supplementary Table 1)
Search	5-6	Present full electronic search strategy for at least one database, including any limits used, such that it could be repeated.	Page 5-6 (Search strategy and selection criteria, Supplementary Table 1)
Study selection	6	State the process for selecting studies (i.e., screening, eligibility, included in systematic review, and, if applicable, included in the meta-analysis).	Page 6 (Data extraction and quality assessment)
Data collection process	6	Describe method of data extraction from reports (e.g., piloted forms, independently, in duplicate) and any processes for obtaining and confirming data from investigators.	Page 6 (Data extraction and quality assessment)

Data items	6-7	List and define all variables for which data were sought (e.g., PICOS, funding sources) and any assumptions and simplifications made.	Page 6-7 (Outcome definition)
Risk of bias in individual studies	6	Describe methods used for assessing risk of bias of individual studies (including specification of whether this was done at the study or outcome level), and how this information is to be used in any data synthesis.	Page 6 (Data extraction and quality assessment)
Summary measures	6-7	State the principal summary measures (e.g., risk ratio, difference in means).	Page 6-7 (Outcome definition)
Synthesis of results	7	Describe the methods of handling data and combining results of studies, if done, including measures of consistency (e.g., I^2) for each meta-analysis.	Page 7 (Statistical analysis)

Section/topic	#	Checklist item	Reported on page #
Risk of bias across studies	7	Specify any assessment of risk of bias that may affect the cumulative evidence (e.g., publication bias, selective reporting within studies).	Page 7 (Statistical analysis)
Additional analyses	7	Describe methods of additional analyses (e.g., sensitivity or subgroup analyses, meta-regression), if done, indicating which were pre-specified.	Page 7 (Statistical analysis)

RESULTS			
Study selection	8	Give numbers of studies screened, assessed for eligibility, and included in the review, with reasons for exclusions at each stage, ideally with a flow diagram.	Page 8 (Search results, Fig 1)
Study characteristics	8	For each study, present characteristics for which data were extracted (e.g., study size, PICOS, follow-up period) and provide the citations.	Page 8 (Patient characteristics, Table 1)
Risk of bias within studies	8-9	Present data on risk of bias of each study and, if available, any outcome level assessment (see item 12).	Page 8-9 (Quality assessment, Fig 2)
Results of individual studies	8	For all outcomes considered (benefits or harms), present, for each study: (a) simple summary data for each intervention group (b) effect estimates and confidence intervals, ideally with a forest plot.	Page 8 (Patient characteristics, Table 1 and Supplementary Table 2)
Synthesis of results	9-11	Present results of each meta-analysis done, including confidence intervals and measures of consistency.	Page 9-11 (TER and MER, Blood gas analysis, Hemorrheology Assessment, Adverse events assessment, Fig 3-7)
Risk of bias across studies	11	Present results of any assessment of risk of bias across studies (see Item 15).	Page 11

			(Publication bias, Fig 8)
Additional analysis	11-12	Give results of additional analyses, if done (e.g., sensitivity or subgroup analyses, meta-regression [see Item 16]).	Page 11-12 (Sensitivity analysis, Fig 9, Table 2)
**DISCUSSION**			
Summary of evidence	12-15	Summarize the main findings including the strength of evidence for each main outcome; consider their relevance to key groups (e.g., healthcare providers, users, and policy makers).	Page 12-15 (Discussion)
Limitations	13-15	Discuss limitations at study and outcome level (e.g., risk of bias), and at review-level (e.g., incomplete retrieval of identified research, reporting bias).	Page 13-15 (limitations)
Conclusions	15	Provide a general interpretation of the results in the context of other evidence, and implications for future research.	Page 15 (Conclusion)
**FUNDING**			
Funding	16	Describe sources of funding for the systematic review and other support (e.g., supply of data); role of funders for the systematic review.	Page 16 (Funding)
